# Phage Therapy Against Antibiotic-Resistant and Multidrug-Resistant Infections Involving Nonhealing Wounds and Prosthetic Joint Infections Associated With Biofilms: A Mini-Review

**DOI:** 10.1155/2024/6252415

**Published:** 2024-11-07

**Authors:** Kashif Haq, Martin Figgitt, David Lee

**Affiliations:** Department of Life Sciences, School of Health Sciences, Birmingham City University, Birmingham B15 3TN, UK

**Keywords:** biofilms, chronic wounds, phage therapy, prosthetic joint infections

## Abstract

Chronic wounds and prosthetic joint infections are difficult to treat and are associated with a high burden of disease and economic cost. The rise of antibiotic resistance and the understanding of biofilm formation has inflamed an already challenging situation. Bacteriophage therapy has been used throughout the last century to treat bacterial infections. However, in the last 10 years, there has been a resurgence in phage therapy as a novel innovative treatment for nonhealing wounds. This mini systemic review assesses relevant clinical studies, case series and trials over 5 years associated with safety, treatment and success rates of phage therapy concerning nonhealing and prosthetic joint infections. A search of PubMed, Web of Science, Cochrane and Clinical Trials.gov databases resulted in 3151 studies, 27 met the criteria, and a total of 152 bacterial infections were treated from 130 individuals. Most common pathogen isolated in wounds was *P. aeruginosa*, and *S. aureus* was mostly associated with prosthetic joint infections. Treatment modalities differed across studies, adverse effects were limited, and success rate was deemed to be 91%.

## 1. Introduction

Wound healing is a complex biological process involving the stages of haemostasis, inflammation, proliferation and maturation [1]. The interplay of these stages has been discussed elsewhere [2]. Chronic wounds (CWs) or nonhealing wounds do not follow the stages of repair in a timely manner and are known not to progress past the inflammation stage [3], resulting in decreased anatomic and functional integrity of the limb. Nonhealing wounds resulting from diabetic foot ulcers, leg ulcers (arterial and venous), postsurgical wounds and pressure ulcers cost the UK National Health Service (NHS) up to three billion pounds per year to treat [4–6]. Bone and periprosthetic joint infections (PJI) are serious infections and complications of arthroplasty surgery [7]. In the UK, it is estimated 2.2% of failed arthroplasty surgeries are due to PJI and the percentage rises to 40% following revision surgery [8, 9], resulting in longer hospital admissions, poor clinical outcomes, increased morbidity, and mortality rates [10–12].

Bacteria within the skin microbiome are known to play a role in healing and impeding wound infections [13]. Once bacteria proliferate the tissue and bacterial load becomes too high, a clinical infection manifests [14]. CWs are polymicrobial in nature, the most common pathogens isolated are *Staphylococcus aureus and Pseudomonas aeruginosa* [15, 16], followed by *Escherichia coli*, *Klebsiella pneumoniae*, *Enterococcus faecalis*, *Acinetobacter baumannii* and *Enterobacter* spp [17]. However, chronic microbiota can vary from patient to patient depending on the type of infection [18, 19]. Predominant pathogens isolated in PJI are usually classified at the time of onset after surgery. Early-onset infection is believed to occur within the first 3 months and can be through cross-contamination during surgery [20]. Most common organisms isolated are *Staphylococci* spp including *S. aureus and S. epidermidis* [21]; however, it is not uncommon to isolate aerobic Gram-negative Bacilli, such as *E. coli* and *P. aeruginosa* through acute haematogenous spread [22]. Delayed infection occurs between 3 and 24 months after arthroplasty, and the major causative organisms are coagulase-negative *staphylococci* (*S. epidermidis, S. caprae* and *S. lugdunensis*) [23, 24] and *Cutibacterium acnes* [25, 26]. Late onset of infection is usually caused by haematogenous spread of an infection or direct contamination and can occur within 12–24 months after the procedure [22, 24].

Biofilms are associated with CW infections and PJIs, and they play a key role in facilitating bacterial growth, induce and invade immune responses, contributing to reduced healing rates and increased inflammation [24–26]. The development of a biofilm in CW and PJI is very similar, and bacteria initially attach themselves to abiotic and biotic surfaces through specific receptor ligands or electrostatic interactions between the bacterium and the surface involved [8, 25, 27–31]. Notably in PJI, this can result in loosening of the implant [8, 22, 32, 33]. Once a biofilm has established itself, it can be difficult to treat and eliminate. Numerous mechanisms, such as heterogeneity of the biofilm, horizontal transference of antimicrobial-resistant (AMR) genes within the biofilm, concentration gradients and diffusional distribution within the exopolymer, all contribute to the resistome of the biofilm [34–40]. Moreover, persister cells, a slow-growing subpopulation of bacteria within a biofilm, demonstrate tolerance to antibiotics phenotypically but not genetically and are known to prolong infection [41–43]. In PJI, bacterial phenotypes can be expressed as small colony variants (SCVs) [23, 44–46] usually characterised by slow growth, metabolism and persistent inflammation [47–49].

Bacteriophages (phages) are viruses, and they can infect and cause lysis of bacteria without causing harm to mammalian cells. They are the most abundant organisms in nature, are ubiquitous within the environment and outnumber bacteria by a factor 10 [50–52]. In the last decade, phage therapy has gained momentum as a tool against multidrug-resistant (MDR) organisms and difficult-to-treat infections [53, 54]. Unlike antibiotics, lytic phages are very specific and have a narrow range of activity, targeting only specific bacteria. Studies have shown they can destroy persister cells and employ depolymerases and other lytic enzymes to destroy exopolysaccharides within the biofilm [55, 56]. Additionally, it can be used with antibiotics [57]. These attributes make phages an attractive antimicrobial agent against MDR infections.

This mini-review of phage therapy for CW and orthopaedic infections was conducted from PubMed and Web of Science databases and Cochrane database of clinical trials, from 2017–2023, using the following terms: phage therapy and infection, phage therapy and wounds.

**Figure 1:**
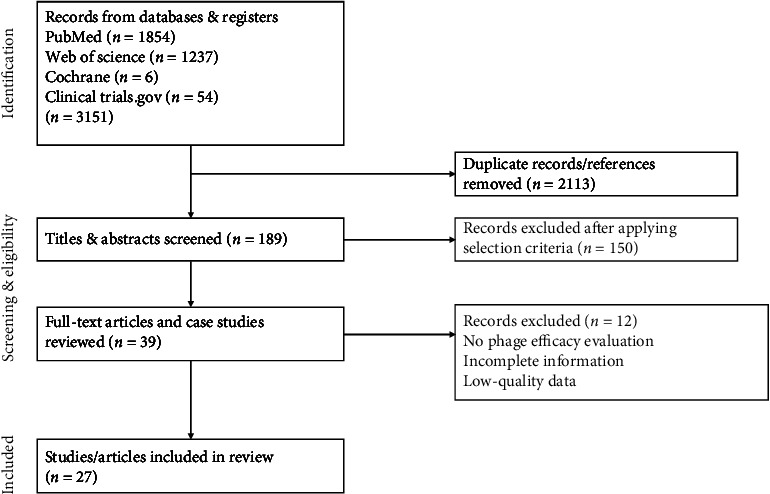
Flow diagram of search methodology undertaken.

All articles were reviewed and to be included, and studies were required to: (1) Report human trials (any patient population or demographics) assessing CW and orthopaedic infections and search terms such as pressure ulcer, diabetic foot ulcer, burn, osteoarticular, implant and prosthetic were accepted. (2) Articles must be in the English language between 2017 and 2023. (3) Treatment regime and outcomes to be reported (no constraints were imposed on modes of phage delivery or dosage regime). Excluded articles included studies involving gastrointestinal, respiratory, genitourinary, pleuro-pulmonary and sepsis-associated phage therapy, preclinical trials, animal trials, in vitro experimentation and records which could not distinguish wound-type infections.  Figure 1 illustrates a flow chart determining search methodology and results.

## 2. Results

A total of 3151 records published between 2017 and 2023 were retrieved. 27 met the eligibility criteria, the majority (25) were case series and/or reports, and the remaining two were case studies. The USA reported most phage-treated infections at 51.8% (*n* = 14), followed by France and Germany (11.1%, *n* = 3), India and Georgia (7.4%, *n* = 2), Australia, Belgium and Italy had one case series each (3.7%).

**Figure 2:**
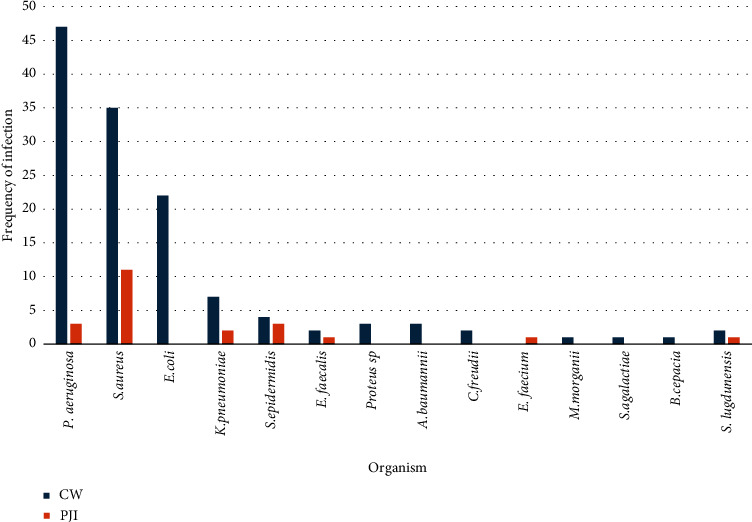
Number of infections caused by organisms resulting from chronic wounds (CWs) and prosthetic joint infections (PJI) mentioned in the articles*. P. aeruginosa* most common pathogen isolated in chronic wounds and *S. aureus* most common pathogen isolated in PJI.

**Figure 3:**
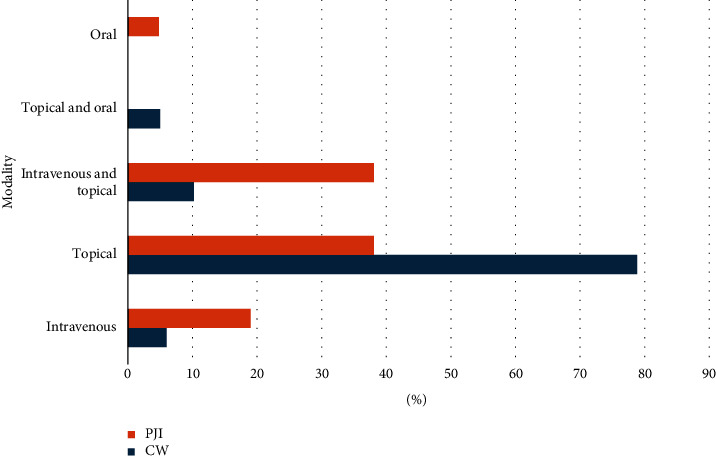
Treatment of chronic wounds (CWs) and prosthetic joint infections (PJIs) through various administrative modalities. Topical treatment was the most common form of modality.

A total of 130 individuals received a single phage or a cocktail of bacteriophages for treatment and 152 bacterial infections in total were treated with phages. Two studies [58, 59] were modified by adding new bacteriophages. 130 wound infections (including ulceration, post-op infection and osteomyelitis) were treated with phages; 22 were treated for PJI (Tables 1 and 2), respectively; and 5.3% of infections were polymicrobial in nature. Majority of infections (Figure 2) in wounds were caused by *Pseudomonas aeruginosa* (36.2%, *n* = 47/130), *S. aureus* (27.0% *n* = 35/130) and *Escherichia coli* (16.9%, *n* = 22/130). Of the data available, not including the study by Patel [71], 49.5% of cases (*n* = 50/101) were applied to MDR bacteria and cocktail phage therapy was used in 65% of those cases (*n* = 17), whereas 35% (*n* = 9) of single-phage was administered. Most common modality of treatment for nonhealing wounds was through topical administration (Figure 3). In this review, the definition included intraoperative, intraarticular and local application modalities (78.8% *n* = 93).

10.2% (*n* = 12) included a joint treatment of topical and intravenous followed by intravenous at 6% (*n* = 7) and topically and orally at 5.8% (*n* = 6), respectively.

In PJI (Figure 3), most common modality was topical and the combination of intravenous and topical at 38.1% (*n* = 8), followed by intravenous (19%; *n* = 4) and orally taken (4.8%; *n* = 1).

Most common organism isolated was *S. aureus* (50% *n* = 11/22), followed by *S. epidermidis* and *P. aeruginosa* (both at 13.6% *n* = 3/22), respectively.

## 3. Discussion

The results of this mini-review demonstrate phage therapy as a potential advocate to treat nonhealing wounds and biofilm-associated infections caused by resistant and nonresistant bacteria. Success rate for PT in this review was calculated at 91%, when compared to similar systemic reviews published over the last few years, and it had a lower success rate than Clarke et al. [84] (96.4%), but higher than Aranaga et al. [85] (85%) and Genevière et al. [86] (71%), respectively. The differences can be attributed to different types of case studies included and the time frame of the review; for example, case reports for all types of PT in English and Spanish were accepted by Aranaga et al. [85] or only bone and joint infections were included over a short or long period of time [86]. In this review, only CW, bone and joint infections involved in biofilm infections were included within a 5-year period. The results are promising; however, the varied heterogeneity between the studies establishing appropriate conclusions is difficult; for example, most case reports and/or case studies [59, 65–68, 70, 72, 73, 75, 76, 81] suggest individuals received phage therapy because of failed antibiotic therapy. The available data implied patients continued with antibiotic therapy while undergoing phage therapy, suggesting that there are unknown complex phage–antibiotic synergistic mechanisms contributing to the elimination of infection [87]. Through an observational point of view, this is a positive outcome for the patient; however, explanations of how infection has been eliminated are rarely explained in scientific terms; for example, there is an evolutionary trade-off between the phage and target bacteria, as previously confirmed [88–90], increasing the sensitivity of antibiotics. Notably, an approach to evolutionary trade-off could advance phage therapy by promoting well-defined phages which have characterised molecular mechanisms correlating to the targeted bacteria. This could explain why monophage therapy is successful in some case studies [66, 78]. Nevertheless, with so many reports from different authors [91–93] on the efficacy of phage therapy, observational findings may contribute strongly towards the safety, efficacy and significance of such treatments.

Phage dose varied between infections; according to the literature review, the dosage was between 10^6^ and 10^9^ PFU/mL (plaque forming unit per millilitre) in CW applications and 10^8^–10^10^ PFU/mL in PJI. Administration, frequency and duration of the therapy differed considerably within the treatment protocols. For example, a single study [74] administered one dose intravenously for 5 days and a second study [73] for 1 day. Regarding topical applications, treatment lasted between 7 days [64] and 7 weeks [66]. Robust data demonstrating optimal dosages for phage therapy between modalities are difficult to compare; moreover, the pharmacokinetics of phage therapy is not fully understood, as phage activity depends up on amplification for efficient lysis to occur. Therefore, parameters such as route of administration, where the infection is and when the phage starts to lyse the target bacteria need to be fully addressed, and this could mean certain infections cannot be appropriately treated with phages alone. Nevertheless, numerous studies have used high doses of phage through topical to nebulisation modalities and hydrogels with no safety or efficacy issues [78, 94]. Moreover, this review found authors had applied topical application to different complex musculoskeletal and PJI resulting in positive prognosis with no or minimum adverse effects, regardless of the underlying medical conditions experienced by the patients [61, 62, 66, 67, 77, 80, 81].

Adverse effect of phage therapy was experienced by 4.6% (*n* = 6) of patients, the most common issue arising from elevated liver function tests (LFT), alanine aminotransferase (ALT) and aspartate aminotransferase (AST); additionally, one case series reported raised tumour necrosis factor (TNF-*α*) [76]. Interestingly, increase in liver enzymes only occurred in PJI involving *S. aureus* and *S. epidermidis* [73, 77, 80, 82], and in one case, PT was discontinued due to hepatomegaly, but the infection was cleared [77]. Rubalskii [70] reported, after successful treatment of a polymicrobial infection consisting of *P. aeruginosa, S. aureus and E. faecium* of a 52-year-old male, the patient passed away 2 months later from an *E. coli* infection. Another study [74] reported the death of a 70-year-old male after completion of phage therapy against *P. aeruginosa*; however, it was deemed that it was not related to PT. A 2-year-old boy passed away during PT against MDR *P. aeruginosa* infection, and treatment did produce a positive outcome through negative blood cultures but was terminated due to decompensation of the patient. Treatment was reinstated; however, patient deteriorated and passed away from septic shock and cardiac arrest, and it is believed this was not associated with PT but a result of other commodities. Ferry et al. [81] reported the death of 2 patients, from non–small-cell lung cancer (NSCLC) and severe acute pancreatitis after the completion of PT, and a positive outcome was reported because microbiological investigations indicated no signs of infection. A death of a patient did occur in the controlled clinical trial of PhagoBurn against *P. aeruginosa* infection [58]; however, the audit determined it was not associated with PT. PhagoBurn was the first randomised control trial registered with the European Clinical Trials database to investigate phage therapy. It consisted of a cocktail of 12 lytic phages (PP1131) targeting *P. aeruginosa* infection in burn wounds. Treatment was through a daily topical modality for 7 and 14 days. Ultimately, the trial was deemed a failure due to the low concentrations of phage cocktail and slow healing rate compared to the control [58].

From the case studies, Patel et al. [71] reported 39 individuals were clear of infection, 7 were partially healed, and 2 died, but it is not clear if this was due to PT. Another study [53] demonstrated that seven patients had complete healing of the wound and no sign of infection by day 21, the remaining patients showed improvement and partial healing of wounds, and both authors indicated topical bacteriophage therapy can be an effective treatment for nonhealing wounds.

## 4. Conclusion

According to this review, phage therapy can be effective and safe against several bacterial species including antibiotic-resistant strains causing wound and biofilm-related infections, with a success rate of 91%. They can be administered through numerous modalities without severe adverse effects. However, success depends on the interdynamics of phage therapy. Effective clinical trials are required to fully evaluate the efficacy of phage therapy, and further studies in formulation and standardisation of such treatments need to be undertaken and unified before they can be fully utilised in a public setting.

## Figures and Tables

**Table 1:** Data selection of selected articles of chronic wounds given phage treatment.

Author(s) and year of publication	Type of infections	Demographics	Organism	Multi–drug-resistant	Phage identification	Dose (PFU/mL) and duration	Application	Clinical outcomes	Adverse effects
Duplessis et al., 2018 [60]	Endovascular infection	2-year-old male	*P. aeruginosa*	Yes	Local phage cocktail adjuvant	3.5 × 10^5^ Not stated	Intravenous	Promising, but patient passed away from heart failure	N/A

Ferry et al., 2018b [61]	Osteomyelitis of the sacroiliac joint	60-year-old male	*P. aeruginosa*	Yes	*Pherecydes* Pharma anti–*P. aeruginosa* phage	1.2–9.7 × 10^8^ for 12 days	Injection	Positive. No sign of infection after 4 weeks	None reported. Patient died from NSCLC

Fish et al., 2018 [62]	Diabetic ulcer on the left third proximal interphalangeal joint—osteomyelitis	48-year-old male	*S. aureus*	No	*Staphylococcu*s phage Sb-1	Not stated, but treatment lasted for 7 weeks	Topical	Positive. Wound healed after 2 months	None reported
	Ulceration on the dorsal left second toe—osteomyelitis	27-year-old female	*S. aureus*	No	*Staphylococcu*s phage Sb-1	Not stated but treatment lasted for 7 weeks	Topical	Positive outcome. Ulcer healed within 5 months	None reported
	Wound on second toe of right foot	74-year-old male	*S. aureus*	No	*Staphylococcu*s phage Sb-1	Not stated but treatment lasted for 7 weeks	Topical	Positive outcome. Wound healed within 2 months	None reported
	Diabetic ulcer on left hallux—osteomyelitis	60-year-old male	*S. aureus*	No	*Staphylococcu*s phage Sb-1	Not stated but treatment lasted for 7 weeks	Topical	Positive outcome. Wound healed within 4 months	None reported
	Diabetic foot ulcer on right third toe becomes gangrenous	61-year-old male	*S. aureus*	No	*Staphylococcu*s phage Sb-1	Not stated but treatment lasted for 7 weeks	Topical	Positive outcome. Wound healed within 2 months	None reported. PT acted as prophylaxis—there was no sign of infection
	Osteomyelitis of left hallux	71-year-old female	*S. epidermidis* *S. lugdunensis*	No	*Staphylococcu*s phage Sb-1		Topical	Positive outcome. Wound healed within 2 months	None reported

Fish et al., 2018b [63]	Osteomyelitis—distal phalangeal	63-year-old female	*S. aureus*	Yes	*Staphylococcu*s phage Sb-1	1 × 10^7^	Injection	Positive. No sign of infection and re-ossification of distal phalangeal	None reported

Morozova et al., 2018 [64]	Multiple diabetic ulcers on left foot	60-year-old male	MRSA	Yes	Bacteriophage preparations by Microgen	1 × 10^6^ for 7 days	Topical	Wound healed within 4 weeks	None reported

Jault et al., 2018 [58]	Wound infection	Trial, 8 males and 4 females	*P. aeruginosa*	No	PhagoBurn (PP1131)	2.14 × 10^7^ for 7 and 14 days	Topical	Negative outcome. Dose too low for effective treatment	None reported

LaVergne et al., 2019 [65]	Craniotomy site infection	77-year-old male	*A. baumannii*	Yes	NMRC phage bank cocktail	2.14 × 10^7^ for 8 days	Intravenous	Positive—no sign of infection	None reported

Gupta et al., 2019 [53]	Chronic ulcer wounds study, 20 people, 12–60 yr. old	Patient 1	*S. aureus*	Yes	Local phage cocktail	1.6 × 10^6^ for 9 days	Topical	Positive—wound healed	None reported
		Patient 2	*S. aureus*	Yes	Local phage cocktail	2.1 × 10^6^ for 9 days	Topical	Positive—wound healed	None reported
		Patient 3	*S. aureus*	Yes	Local phage cocktail	1.8 × 10^6^ for 9 days	Topical	Positive—wound healed	None reported
		Patient 4	*S. aureus*	Yes	Local phage cocktail	1.2–1.6 × 10^6^ for 13 days	Topical	Positive—wound healed	None reported
		Patient 5	*S. aureus*	Yes		3.0–1.4 × 10^6^ for 13 days	Topical	Positive—wound healed	None reported
		Patient 6	*E. coli*	Yes	Local phage cocktail	3.1–2.3 × 10^6^ for 13 days	Topical	Positive—wound healed	None reported
		Patient 7	*E. coli*	Yes	Local phage cocktail	2.1 × 10^6^ for 9 days	Topical	Positive—wound healed	None reported
		Patient 8	*E. coli*	Yes		3.0–1.4 × 10^6^ for 13 days	Topical	Positive—wound healed	None reported
		Patient 9	*E. coli*	Yes	Local phage cocktail	2.1 × 10^6^ for 9 days	Topical	Positive—wound healed	None reported
		Patient 10	*E. coli*	Yes	Local phage cocktail	3.2–2.0 × 10^6^ for 13 days	Topical	Positive—wound healed	None reported
		Patient 11	*E. coli*	Yes		2.8–1.5 × 10^6^ for 13 days	Topical	Positive—wound healed	None reported
		Patient 12	*P. aeruginosa*	Yes	Local phage cocktail	2.7–1.2 × 10^6^ for 13 days	Topical	Positive—wound healed	None reported
		Patient 13	*P. aeruginosa*	Yes	Local phage cocktail	3.4–2.0 × 10^6^ for 13 days	Topical	Positive—wound healed	None reported
		Patient 14	*P. aeruginosa*	Yes		1.6 × 10^6^ for 9 days	Topical	Positive—wound healed	None reported
		Patient 15	*P. aeruginosa*	Yes	Local phage cocktail	2.9–1.3 × 10^6^ for 13 days	Topical	Positive—wound healed	None reported
		Patient 16	*P. aeruginosa*	Yes	Local phage cocktail	1.6 × 10^6^ for 9 Days	Topical	Positive—wound healed	None reported
		Patient 17	*P. aeruginosa*	Yes		2.1 × 10^6^ for 9 days	Topical	Positive—wound healed	None reported
		Patient 18	*P. aeruginosa*	Yes	Local phage cocktail	2.3–1.1 × 10^6^ for 13 days	Topical	Positive—wound healed	None reported
		Patient 19	*P. aeruginosa*	Yes	Local phage cocktail	2.1 × 10^6^ for 9 days	Topical	Positive—wound healed	None reported
		Patient 20	*P. aeruginosa*	Yes		2.5 × 10^6^ for 9 days	Topical	Positive—wound healed	None reported

Nir-Paz et al., 2019 [66]	Left tibial infection	42-year-old male	*A. baumannii* *K. pneumoniae*	Yes	*ϕ*AbKT21phi3 *ϕ*KpKT21phi1	5 × 10^7^ for 5 days	Intravenous	Positive outcome. Healing of wound, no positive cultures after 8 months	None reported

Onsea et al., 2019 [67]	Osteomyelitis of the pelvis	Patient	*P. aeruginosa and S. epidermidis*	Yes	Cocktail BCF1	2 × 10^7^ for 7 days	Intraoperative—topical	Positive. Patient recovered	None reported
	Osteomyelitis of the femur	Patient	*P. aeruginosa and S. epidermidis*	Yes	Cocktail BCF1 and PYO phage	2 × 10^7^ for 10 days	Intraoperative—topical	Positive. Patient recovered	None reported
	Postoperative infection of wound	Patient	*S. agalactiae and S. aureus*	Yes	Cocktail BCF1	2 × 10^7^ for 9 days	Intraoperative—topical	Positive. Patient recovered	None reported
	Infected abscess	Patient	*E. faecalis*	Yes	Cocktail BCF1	2 × 10^7^ for 7 days	Intraoperative—topical	Positive	None reported
	Osteomyelitis of the tibia	60-year-old female	*S. aureus*	Yes	Local phage adjuvant	Not stated	Topical—intraoperative	Positive. Wound healed	None reported

Tkhilaisshvili et al., 2019 [68]	Osteomyelitis of the knee and femur	80-year-old female	*P. aeruginosa and S. epidermidis*	Yes No	Local phage adjuvant	1 × 10^8^ for 5 days	Local application	Positive. Negative cultures	None reported

Nadareishvili et al., 2020 [69]	Osteomyelitis of the sternum	74-year-old male	*S. aureus*	No	Staphylococcal and SES bacteriophage	Not stated, but treatment was for 20 days	Topical and oral	Positive outcome. Wound healed	None reported
	Ulcer and osteomyelitis of the foot	69-year-old male	*B. cepacia* *E. faecalis and S. aureus*	Yes	Staphylococcal and intesti bacteriophage	Not stated but treatment was for 20 days	Topical and oral	Positive outcome. Ulcer healed. Patient improved	None reported
	Osteomyelitis of right tibia	60-year-old female	*S. aureus*	Yes	SES and intesti bacteriophage	Not stated but treatment was for 20 days	Topical and oral	Positive outcome. Ulcer healed. Patient improved	None reported
	Postsurgical wound	68-year-old male	*P. aeruginosa*	Yes	PYO and intesti bacteriophage	Not stated but treatment was for 3 months	Topical and oral	Positive outcome. Wound healed. Patient improved	None reported

Rubalskii et al., 2020 [70]	Vascular graft infection	40-year-old male	*S. aureus*	No	*Staphylococcus* phage CH1	1 × 10^9^ 2 days	Local application	Positive outcome. *S. aureus* not detected	None reported
	Sternal wall after cardiac surgery	66-year-old male	*E. coli*	Yes	*Escherichia phage ECD7 and V18*	4 × 10^10^ 1 day	Local application	Positive. No *E. coli* detected	None reported
	Sternal wound abscesses	12-year-old male	*P. aeruginosa*	Yes	*Pseudomonas phage PA5 and PA10*	4 × 10^10^ 1 day	Local application	Positive outcome No *P. aeruginosa*	None reported

Patel et al., 2021 [71]	Range of chronic wound infections of the lower extremities and infected ulcers	Study, 48 patients. 34 males and 14 females	*A. baumannii (1) E. coli (15), C. freundii (2) K. pneumoniae (6), Proteus* spp *(3) S. aureus (15), P. aeruginosa (18), M. morganii (1)*	Not known	Local phage cocktail adjuvant	1 × 10^9^ 5–7 applications at 12 h	Topical	Positive, 39 patients clear of infection Remaining had slower recovery rate, due to poor hygiene	None reported

Khatami et al., 2021 [72]	Osteomyelitis of the foot	7-year-old female	*P. aeruginosa*	Yes	Pa14NP*ϕ*PASA16	1 × 10^11^ once a day for 7 days	Intravenous	Positive outcome	None reported

Doub et al., 2023 [73]	Osteomyelitis of the knee	Patient 7	*S. aureus*	Yes	*Staphylococcus* phage Mallokai	1 × 10^10^ for 4 days	Intravenous intraarticular	Positive outcome	None reported
	Osteomyelitis of knee and hip	Patient 8	*S. aureus*	Yes	*Staphylococcus* phage Mallokai	1 × 10^10^ for 1–3 days	Intravenous intraarticular	Positive outcome	Raised LFT levels
	Osteomyelitis of the knee	Patient 9	*S. aureus*	Yes	SaGR51*ϕ*1	1 × 10^10^ for 1–3 days	Intravenous intraarticular	Positive outcome	Raised LFT levels
	Osteomyelitis of the knee	Patient 10	*S. lugdunensis*	No	*Staphylococcus* phage Mallokai	1 × 10^10^ for 4–5 days	Intravenous intraarticular	Positive outcome	None reported

Abbreviations: ALT, alanine aminotransferase; AST, aspartate aminotransferase; LFT, liver function test; NSCLC, non–small-cell lung cancer.

**Table 2:** Data selection of selected articles of PJIs treated with phage therapy.

Author(s) and year of publication	Type of infection	Demographics	Organism	Multidrug-resistant	Phage identification	Dose (PFU/mL) and duration	Application	Clinical outcomes	Adverse effects
Ferry et al., 2018 [74]	Prosthetic joint infection	80-year-old female	*S. aureus*	No	Local phage adjuvant	6 × 10^10^ for 1 day	Intravenous	Positive outcome	None reported

Aslam et al., 2020 [75]	Prosthetic knee joint infection	61-year-old female	*S. aureus*	No	*Staphylococcu*s phage AB-SA01	3 × 10^9^ for 14 days	Intravenous	Positive outcome for 2 weeks, but additional antibiotics and phage therapy need at day 17	None reported

Cano et al., 2020 [76]	Prosthetic knee joint infection	62-year-old male	*K. pneumoniae*	Yes	KpJH46*ϕ*2	6.3 × 10^10^ (40 doses)	Peripheral intravenous	Positive outcome	Raised TNF-*α* levels. Pruritus of lower right leg

Doub et al., 2020 [77]	Prosthetic knee joint infection	72-year-old male	*S. aureus*	Yes	SaGR51*ϕ*1	2.7 × 10^9^ for 3 days	Intraoperative	Positive. Infection cleared—PT discontinued	Liver enzymes raised—hepatomegaly

Ferry et al., 2020 [78]	Prosthetic knee joint infection	80-year-old male	*S. aureus*	No	PhagoDAIR PP1493, PP1815, PP1957	1 × 10^9^ 1 day	Intraoperative	Positive. Patient recovered	None reported
	Prosthetic knee joint infection	84-year-old male	*S. aureus*	No	PhagoDAIR PP1493, PP1815, PP1957	1 × 10^9^ 1 day	Intraoperative	Positive. Patient recovered	None reported
	Prosthetic knee joint infection	80-year-old female	*S. aureus*	Yes	PhagoDAIR PP1493, PP1815, PP1957	1 × 10^9^ 1 day	Intraoperative	Not fully known, transfemoral amputation performed. Polymicrobial infection	None reported

Ferry et al., 2020b [79]	Knee megaprosthesis infection	49-year-old male	*S. epidermidis*	No	PP1493 and PP1815	1.7 × 10^8^	Topical	Positive outcome	None reported

Rubalskii et al., 2020 [70]	Prosthetic infection	52-year-old male	*E. faecium, P. aeruginosa, and S. aureus*	Yes	*Enterococcus* Phage Enf1, *Pseudomonas* phage PA10 and *Staphylococcu*s phage CH1	1 × 10^8^ 1 × 10^8^ 1 × 10^8^	Orally Local application	Positive, pathogens not detected	None reported. Patient passed away 2 months later from a new *E.coli infection*

Doub et al., 2021 [80]	Prosthetic knee infection	79-year-old female	*S. epidermidis*	No	Bacteriophage adjuvant	2 × 10^10^ for 1 day	Topical Intravenous	Positive. Patient recovered	Raised ALT and AST levels

Ferry et al., 2021 [81]	Prosthetic knee joint infection	88-year-old male	*P. aeruginosa*	No	PhagoDAIR	1 × 10^9^ 1 day	Intravenous	Positive. Patient recovered	None reported, but died of severe acute pancreatitis

Doub et al., 2022 [73]	Prosthetic knee and femoral lateral plate infection	70-year-old female	MRSA	Yes	Mallokai	1 × 10^9^–2 × 10^8^ 3 doses	Intraoperatively and intravenous	Positive. No sign of infection	Raised LFT, mild transaminitis

Schoeffel et al., 2022 [59]	Prosthetic knee infection	64-year-old female	*S. aureus*	Yes	Adaptive phage therapeutics (SaWIQ0488*ϕ*1)	1.2 × 10^9^ for 3 days	Intraoperatively	Infection cleared, but returned once antibiotics were stopped	None reported

Doub et al., 2023 [82]	Prosthetic knee infection	Patient 1	*E. faecalis*	No	*Enterococcus* phage EF1	1 × 10^10^ for 1–5 days	Intravenous intraarticular	Positive outcome	None reported
	Prosthetic shoulder infection	Patient 2	*K. pneumoniae*	Yes	*Klebsiella* phage KP1 and 2	1 × 10^10^ for 2–4 days	Intravenous intraarticular	Positive outcome	None reported
	Prosthetic knee infection	Patient 3	*S. epidermidis*	No	*Staphylococcus* phage PM448	1 × 10^10^ for 1–5 days	Intravenous intraarticular	Infection cleared but arthrodesis to secondary joint	Raised LFT levels
	Prosthetic knee infection	Patient 4	*S. lugdunensis*	No	*Staphylococcus* phage Mallokai	1 × 10^10^ for 1 day	Intravenous intraarticular	Positive outcome	None reported
	Prosthetic knee infection	Patient 5	*S. aureus*	Yes	*Staphylococcus* phage Mallokai	1 × 10^10^ for 2–4 days	Intravenous intraarticular	Positive outcome	Raised LFT levels
	Prosthetic knee and hip infection	Patient 6	*S. aureus*	Yes	SaW1Q0488*ϕ*1	1 × 10^10^ for 1–4 days	Intravenous intraarticular	Positive outcome	Increased LFT levels

Cesta et al., 2023 [83]	Chronic hip prosthetic joint infection	62-year-old female	*P. aeruginosa*	Yes	Pa53	1 × 10^8^ for 15 days	Intravenous	Positive outcome	None reported

Abbreviations: ALT, alanine aminotransferase; AST, aspartate aminotransferase; LFT, liver function test; NSCLC, non–small-cell lung cancer.

## Data Availability

All data generated or analysed during the study are included within this article.
